# Nutritional Composition and Antioxidant Capacity in Edible Flowers: Characterisation of Phenolic Compounds by HPLC-DAD-ESI/MS^n^

**DOI:** 10.3390/ijms16010805

**Published:** 2014-12-31

**Authors:** Inmaculada Navarro-González, Rocío González-Barrio, Verónica García-Valverde, Ana Belén Bautista-Ortín, María Jesús Periago

**Affiliations:** Department of Food Technology, Food Science and Nutrition, Faculty of Veterinary Sciences, Regional Campus of International Excellence “Campus Mare Nostrum”, University of Murcia, Espinardo 30071 (Murcia), Spain; E-Mails: inmanago@yahoo.es (I.N.-G.); rgbarrio@um.es (R.G.-B.); verogv@um.es (V.G.-V.); abautista@um.es (A.B.B.-O.)

**Keywords:** edible flowers, nutritional value, phenolic compounds, HPLC-DAD-ESI/MS^n^, TEAC (Trolox Equivalent Antioxidant Capacity), ORAC (Oxygen Radical Absorbance Capacity)

## Abstract

Edible flowers are commonly used in human nutrition and their consumption has increased in recent years. The aim of this study was to ascertain the nutritional composition and the content and profile of phenolic compounds of three edible flowers, monks cress (*Tropaeolum majus*)*,* marigold (*Tagetes erecta*) and paracress (*Spilanthes oleracea*), and to determine the relationship between the presence of phenolic compounds and the antioxidant capacity. Proximate composition, total dietary fibre (TDF) and minerals were analysed according to official methods: total phenolic compounds (TPC) were determined with Folin-Ciocalteu’s reagent, whereas antioxidant capacity was evaluated using Trolox Equivalent Antioxidant Capacity (TEAC) and Oxygen Radical Absorbance Capacity (ORAC) assays. In addition, phenolic compounds were characterised by HPLC-DAD-MS^n^. In relation to the nutritional value, the edible flowers had a composition similar to that of other plant foods, with a high water and TDF content, low protein content and very low proportion of total fat—showing significant differences among samples. The levels of TPC compounds and the antioxidant capacity were significantly higher in *T. erecta*, followed by *S. oleracea* and *T. majus*. Thirty-nine different phenolic compounds were tentatively identified, with flavonols being the major compounds detected in all samples, followed by anthocyanins and hydroxycynnamic acid derivatives. In *T. erecta* small proportions of gallotannin and ellagic acid were also identified.

## 1. Introduction

Edible flowers have been eaten as part of human nutrition since ancient times, as they are considered plant foods with medicinal properties and hence beneficial effects for human health. Their consumption has been reported for centuries and includes flowers of different species—like rose, violet, jasmine, monks cress, *Prunus* and flower of Jamaica—that are consumed as ingredients in different meals, salads, foodstuffs and drinks. In European countries, the most common application of flowers in human nutrition is the preparation of hot beverages (tisane or infusion), giving several types of flower teas, which are drunk with the aim of providing wellness due to the medicinal properties of each kind of flower [1,2,3,4]. From a dietary point of view, a great advantage of teas made with edible flowers is the fact that they do not contain caffeine, whereas many types of tea contain stimulant drugs such as the alkaloid xanthenes caffeine and theobromine [4].

Not all flowers are edible; to be included in a human diet, flowers have to be non-toxic and innocuous (considering the presence of biological and chemical hazards) and have nutritional properties [3,4]. Some flower species have toxic substances that could affect their nutritional properties, such as trypsin inhibitors, or—even worse—cause severe damage to consumers; for example, those that contain hemaglutinnins, oxalic acid, cyanogenic glycosides or alkaloids [2,3,5]. These flowers must be considered inedible and hence cannot be commercialized or included in the human diet. However, for those that are recognised as edible, it is very important to know their nutritional composition and other characteristics of interest in human nutrition. Related to their proximate composition, the main component of edible flowers is water (more than 80%) and their protein and fat contents are considered to be low, with different amounts of total carbohydrates, dietary fibre and minerals according to the kind of flower [2,3,5,6,7]. Other properties of flowers are related to the content of bioactive compounds like carotenoids, phenolic compounds and essential oils, which provide a wide range of functional properties.

Edible flowers contain phenolic compounds with different chemical structures, mainly phenolic acids, flavonols and anthocyanins, which provide antioxidant capacity and protect against the damage induced by free radicals [8,9,10]; these have been positively related to human metabolism [11]. However, there is much controversy as to whether polyphenols retain their antioxidant features *in vivo*, following ingestion, because the circulating concentrations of polyphenols normally do not exceed the low micromolar range, and hence their real contribution to the overall antioxidant capacity appears to be negligible [12]. In addition to the phenolic compounds, carotenes (which provide colour) [13], isothiocyanates [14], essential oils (the main component of the flower smell) [15] and circular plant peptides called cyclotides [16] could exert other pharmacological effects.

*Tropaeolum majus* (monks cress) is distributed around the world, and several pharmacological and experimental studies have provided information about its bioactivity, including antibacterial activity against infections [17], *in vitro* and *in vivo* antitumor activity [14], antithrombotic activity [18] and diuretic and hypotensive effects [19,20]. In addition, flowers of the genera *Spilanthes* and *Tagetes* also exhibit several pharmacological effects, such as vasodilatation, immunomodulation, diuretic, antibacterial and anti-inflammatory activities and hypotensive and analgesic properties [21,22,23,24,25], and hence they have been used for a long time as herbal remedies in human nutrition [24]. Taking into consideration that edible flowers can be incorporated into the human diet and are an important source of antioxidant bioactive compounds, the aim of this study was to ascertain the nutritional composition, total phenolic content and phenolic profile of three species of edible flower: monks cress (*Tropaeolum majus*), marigold (*Tagetes erecta*) and paracress (*Spilanthes oleracea*). In order to determine their potential beneficial effects on human metabolism, the relationship between the presence of antioxidant compounds and the antioxidant capacity was also determined.

## 2. Results and Discussion

### 2.1. Nutritional Composition

The proximate compositions of the edible flowers of *T. majus*, *T. erecta* and *S. oleracea* are shown in Table 1. As described in other vegetables or plant foods, the edible flowers showed a high water content, that of *T. majus* being the highest. Total carbohydrates were the most abundant macronutrient, with significant differences (*p* < 0.05) among samples. They were comprised of digestible carbohydrates, simple sugars and indigestible carbohydrates, the latter mainly represented by total dietary fibre (TDF). Notably, TDF was the main component of the total carbohydrates, as simple sugars ranged from 2.63% to 4.95% and TDF from 4.51% to 10.11%, with significant differences (*p* < 0.05) among the three species. The protein and ash contents were lower than 3% and 1.5%, respectively, and differed significantly among the three species (*p* < 0.05). However, the total lipid content was lower than 0.5% and no significant differences were observed for this parameter. All samples showed a very low energetic value, less than 30 kcal/100 g; the lowest caloric value was for *T. majus*, due to the fact that it had the lowest carbohydrate and lipid contents.

**Table 1:** Proximal composition of different edible flowers ^1^.

Parameter	*Tropaeolum majus*	*Tagetes erecta*	*Spilanthes oleracea*
Moisture (%)	89.32 ± 0.16 ^a^	83.39 ± 0.17 ^b^	81.74 ± 0.13 ^c^
Total carbohydrates (%)	7.14 ± 0.87 ^c^	14.15 ± 1.24 ^a^	13.56 ± 0.79 ^b^
TDF (%)	4.51 ± 0.52 ^b^	9.20 ± 0.04 ^a^	10.11 ± 0.41 ^a^
Protein (%)	1.99 ± 0.06 ^b^	1.32 ± 0.01 ^b^	2.84 ± 0.11 ^a^
Fat (%)	0.33 ± 0.03 ^a^	0.32 ± 0.02 ^a^	0.41 ± 0.03 ^a^
Ash (%)	0.63 ± 0.01 ^c^	0.80 ± 0.05 ^b^	1.44 ± 0.02 ^a^
Energy (kcal/100 g)	21.44 ± 0.89 ^b^	28.02 ± 1.1 ^a^	28.84 ± 1.20 ^a^

^1^ Data are expressed as percentage of fresh weight (mean value ± standard deviation). ^a–c^ Means with different letters within a row are significantly different (*p* < 0.05). TDF, total dietary fibre.

The mineral composition of the edible flowers, expressed on a fresh weight basis, is shown in Table 2. The most abundant mineral elements in *T. majus* were zinc, iron, copper, manganese, strontium and potassium; in *T. erecta* iron, strontium, zinc, manganese and potassium; and in *S. oleracea* iron, strontium, manganese, zinc and potassium. From a nutritional point of view, it is noteworthy that the edible flowers of the three species showed higher concentrations of potassium than of sodium; however, the calcium concentration was very low in the flowers, especially in *T. majus.* Other elements, namely, phosphorus, sodium, magnesium and sulphur, were detected at low concentrations, without significant differences among the species, whereas beryllium, bismuth, cadmium, chrome, cobalt, nickel, selenium and vanadium were detected at very low concentrations and have not been included in the table.

**Table 2:** Mineral composition of the edible flowers ^1^.

Mineral	*Tropaeolum majus*	*Tagetes erecta*	*Spilanthes oleracea*
Ca (mg/100 g)	0.055 ± 0.007 ^a^	0.110 ± 0.042 ^a^	0.105 ± 0.035 ^a^
Cu (mg/100 g)	0.472 ± 0.020 ^a^	0.104 ± 0.025 ^b^	0.165 ± 0.057 ^b^
Fe (mg/100 g)	0.551 ± 0.074 ^a^	1.026 ± 0.052 ^a^	1.500 ± 0.540 ^a^
K (mg/100 g)	0.225 ± 0.007 ^a^	0.215 ± 0.007 ^a^	0.355 ± 0.007 ^b^
Mg (mg/100 g)	0.035 ± 0.007 ^a^	0.060 ± 0.00 ^a^	0.06 ± 0.028 ^a^
Mn (mg/100 g)	0.397 ± 0.026 ^a^	0.303 ± 0.027 ^a^	0.555 ± 0.239 ^a^
Na (mg/100g)	0.010 ± 0.00 ^a^	0.015 ± 0.007 ^a^	0.010 ± 0.00 ^a^
P (mg/100 g)	0.050 ± 0.000 ^a^	0.065 ± 0.007 ^a^	0.080 ± 0.020 ^a^
S (mg/100 g)	0.040 ± 0.000 ^a^	0.045 ± 0.007 ^a^	0.060 ± 0.014 ^a^
Sr (mg/100 g)	0.388 ± 0.002 ^a^	1.017 ± 0.470 ^a^	0.897 ± 0.328 ^a^
Zn (mg/100 g)	0.660 ± 0.064 ^a^	0.568 ± 0.093 ^a^	0.543 ± 0.144 ^a^

^1^ Data are expressed as mg/100 g of fresh weight (mean value ± standard deviation). ^a,b^ Means with different letters within a row are significantly different (*p* < 0.05).

In short, the nutritional composition of these edible flowers is not too different from that of other edible flowers [5,7], herbs [26], vegetables, such as *Spinacea oleracea* [27], *Brassica* spp. [28], *Amaranthus* spp. [29] and *Cucurbita* spp. [27], and edible wild green vegetables [30].

### 2.2. Total Penolic Compounds and Their Identification by HPLC-DAD-MS^n^

Table 3 shows the Total Phenolic Compounds (TPC) concentration and the antioxidant capacity of the edible flowers. The TPC was evaluated using the Folin-Ciocalteu assay, which is considered a fast and reliable way to quantify phenolics in foods [31]. The highest TPC concentration was found in *T. erecta* (26.63 mg GAE/g), followed by *T. majus* and *S. oleracea*, which presented the lowest TPC (6.64 mg GAE/g). These results are within the range reported in the scientific literature for the TPC of edible flowers [1,7,32,33,34], although there is high variability in the content of phenolic compounds according to the species.

**Table 3:** Folin total phenolic compounds (TPC), oxygen radical absorbance capacity (ORAC) and Trolox equivalent antioxidant capacity (TEAC) in edible flowers ^1^.

Parameters	*Tropaeolum majus*	*Tagetes erecta*	*Spilanthes oleracea*
TPC (mg GAE/g)	12.95 ± 2.21 ^b^	26.63 ± 4.22 ^a^	6.64 ± 0.45 ^c^
ORAC (µmol TE/g)	47.84 ± 0.80 ^b^	266.11 ± 55.9 ^a^	10.82 ± 0.53 ^c^
TEAC (µmol TE/g)	9.51 ± 0.10 ^a^	66.22 ± 1.10 ^b^	5.52 ± 0.13 ^c^

^1^ Data are expressed on a fresh weight basis, as mean value ± standard deviation. ^a–c^ Means with different letters within a row are significantly different (*p* < 0.05). GAE, gallic acid equivalents; TE, Trolox equivalents.

**Figure 1:**
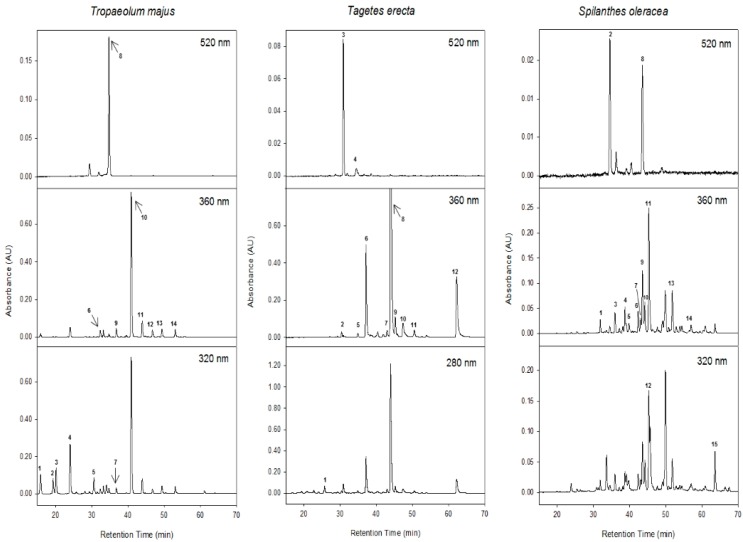
Chromatograms of the edible flowers recorded at 520, 360, 320 and 280 nm. Peak numbers are listed in Table 4.

The HPLC-DAD-MS^n^ analysis of the edible flowers allowed the characterisation of 39 different compounds, including anthocyanins, flavonols, hydroxycinnamic acid derivatives, hydrolysable tannins and phenolic acids. Table 4 summarises the tentative characterisation of these compounds according to their absorbance and mass spectra, based on previously reported data [35,36,37,38,39,40,41]. Comparison with authentic standards, when possible, was used to confirm the identity of some compounds to support the tentative identification. The chromatographic separations are shown in Figure 1 where the compounds identified are labelled as peaks 1 to 15 in *T. majus*, 1 to 12 in *T. erecta* and 1 to 15 in *S. oleracea* flowers, following the elution order in the HPLC and recorded at 520, 360, 320 and 280 nm.

**Table 4:** HPLC-DAD-ESI/MS^n^ analysis of phenolic compounds detected in edible flowers (*T. majus*, *T. erecta* and *S. oleracea*). Retention times (Rt), wavelengths of maximum absorption (λ_max_) and mass spectra. * identification in positive mode.

Sample	Peak	Rt (min)	Tentative Identification	λ_max_ (nm)	[M-H]^−^ (*m*/*z*)	MS^2^ (*m*/*z*)
*Tropaeolum majus*	1	15.8	3-*O*-caffeoylquinic acid	325	353	191, 179
	2	19.3	cis-3-*O*-*p*-coumaroylquinic acid	305	337	163, 173
	3	20.1	trans-3-*O*-*p*-coumaroylquinic acid	310	337	163, 173
	4	24.0	5-*O*-caffeoylquinic acid	325	353	191, 179
	5	30.6	trans-5-*O*-*p*-coumaroylquinic acid	311	337	191, 163
	6	32.3	Myricetin-3-*O*-sophoroside	354	641	317, 461, 479
	7	33.2	cis-5-*O*-*p*-coumaroylquinic acid	305	337	191, 163
	8	34.7	Pelargonidin-3-*O*-sophoroside	502	595 *	271, 415
	9	36.8	Quercetin-3-*O*-sophoroside	353	625	301, 445
	10	40.9	Kaempferol-3-*O*-sophoroside	347	609	285, 429
	11	43.9	Kaempferol-3-*O*-acetyl-sophoroside	348	651	471, 285, 489
	12	46.8	Quercetin-*O*-acetylhexoxide	355	505	301, 463
	13	49.4	Kaempferol-3-*O*-hexoside	348	447	285
	14	53.1	Kaempferol-*O*-acetylhexoxide	348	489	285
*Tagetes erecta*	1	25.7	Gallotannin	277	797	645, 627, 493
	2	30.4	Laricitrin-di-hexoside	356	655	493, 331
	3	30.8	Cyanidin-di-hexoside	518	611 *	449, 287
	4	34.9	Delphinidin-3-*O*-hexoside	516	465 *	303
	5	35.7	Myricetin-hexoside	357	479	317
	6	37.1	Laricitrin-hexoside	358	493	331
	7	42.9	Ellagic acid	365	301	257, 229
	8	43.9	Laricitrin-hexoside	354	493	331
	9	45.2	Laricitrin-hexoside	352	493	331
	10	47.3	Myricetin	360	317	299, 271, 167
	11	50.5	Isorhamnetin-3-*O*-hexoside	354	477	315
	12	62.2	Laricitrin	364	331	316
*Spilanthes oleracea*	1	33.6	Quercetin-deoxyhexoside-di-hexoside	353	771	625, 446, 301
	2	34.5	Cyanidin-3-*O*-glucoside	517	449 *	287
	3	35.9	Quercetin-dihexoside	354	625	301, 463
	4	38.1	Quercetin-rhamnosyl-hexoside	356	609	447, 463, 301
	5	38.7	Quercetin-rhamnosyl-rutinoside	355	755	609, 301
	6	42.3	Quercetin-3-*O*-rutinoside	356	609	301
	7	43.1	Quercetin-3-*O*-glucoside	353	463	301
	8	43.5	Delphinidina-3-*O*-glucuronide	517	479 *	303
	9	43.6	Quercetin-3-*O*-glucuronide	354	477	301
	10	44.2	Quercetin-acetylhexose-deoxyhexoside	352	651	609, 505, 447, 301
	11	45.3	Quercetin-acetyl dihexoside	354	667	625, 301
	12	49.9	dicaffeoylquinic acid	330	515	353
	13	51.8	Quercetin-acetyl hexoside	356	505	463, 301
	14	57.0	Quercetin-diacetyl hexoside	353	547	505, 463, 301
	15	63.6	Caffeoylquinic acid dihexose derivative	326	677	515, 353

**Figure 2:**
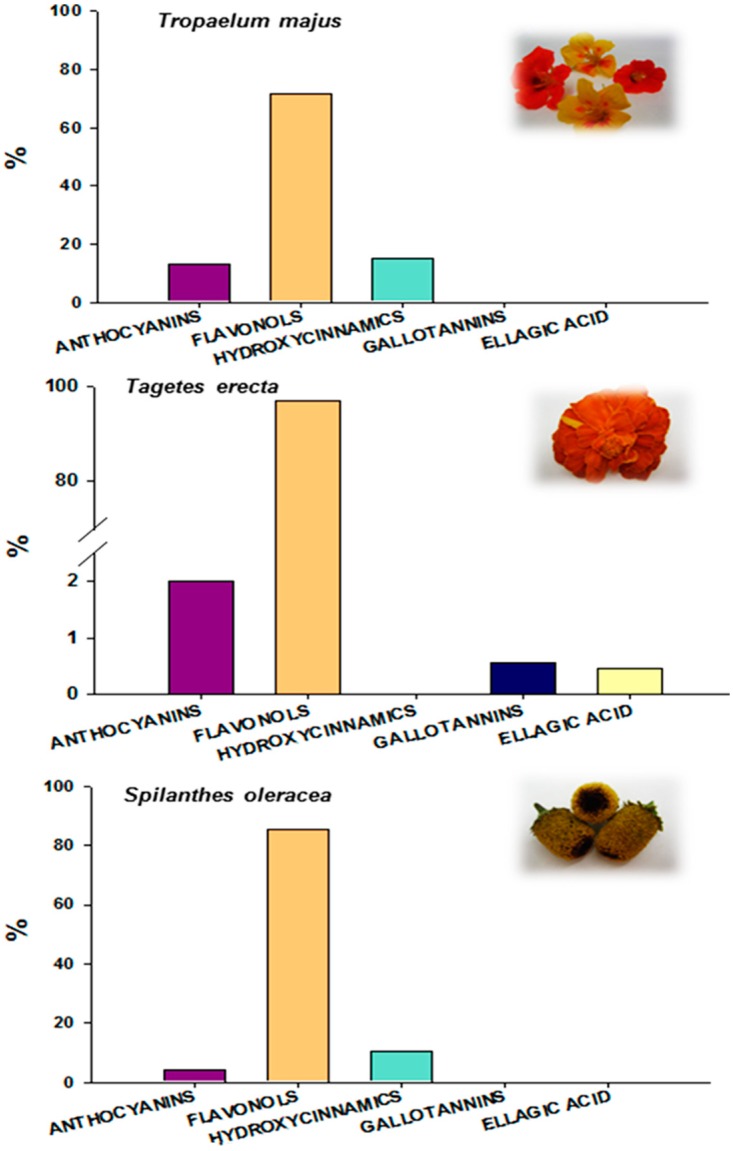
Identification of the edible flowers and the proportions of the individual phenolic compounds as part of the total compounds identified by HPLC-DAD-ESI/MS^n^.

The main phenolic compounds detected in the methanolic extract of *T. majus* are shown in Figure 1 and Figure 2 and Table 4, and were identified as flavonol-glycosides and hydroxycinnamic acid derivatives (581 and 124 µg/g, respectively, data not shown). In addition one anthocyanin was also characterised (105.8 µg/g, data not shown). Peaks 1–5 and 7 had UV spectra with λ_max_ between 305 and 325 nm, characteristic of hydroxycinnamic acids. The mass spectral analysis revealed that they had a negatively charged quasi-molecular ion ([M-H]^−^) at *m*/*z* 353 and 337 (Table 4 and Figure 1). By comparison of their fragmentation pattern with spectrometric characteristics previously reported in the literature, these peaks were identified as isomeric forms of caffeoylquinic acid and *p*-coumaroylquinic acid [36,37,38,40]. A total of seven flavonol-glycosides were also identified in the flowers of *T. majus* according to their UV spectra, with λ_max_ between 347 and 355 nm. Peaks 6, 9 and 10 had an [M-H]^−^ at *m*/*z* 641, 625 and 609, respectively. The MS^2^ analyses showed fragment ions corresponding to the loss of 180 amu [M-H-180]^−^, which is characteristic of the loss of the terminal glucose from a sophorosyl moiety [41]. Other fragments at *m*/*z* 317, 301 and 285 correspond to the mass of myricetin, quercetin and kaempferol (*i.e.*, the aglycons without the sophorosyl moiety), respectively, indicating that peaks 6, 9 and 10 are myricetin-3-*O*-sophoroside, quercetin-3-*O*-sophoroside and kaempferol-3-*O*-sophoroside, respectively. The loss of 180 amu was also observed for peak 11, which allowed the presence of a sophorosyl moiety in the structure to be determined. This peak displayed a UV spectrum similar to that of peak 10; however, peak 11 had an [M-H]^−^ at *m*/*z* 651, indicating that this peak is kaempferol-3-*O*-acetylsophoroside.

Peak 8 had a characteristic UV spectrum with λ_max_ at 502 nm, corresponding with an anthocyanin glycoside, and a positively charged quasi-molecular ion ([M]^+^) at *m*/*z* 595, while the MS^2^ spectra showed two fragments, at *m*/*z* 415 and 271. This peak produced a similar loss of 180 amu [M+H-180]^+^, indicating the presence of a sophoroside moiety, as described above. The second fragment at *m*/*z* 271 corresponds to the mass of pelargonidin aglycone [M-324]^+^, indicating that this peak is pelargonidin-3-*O*-sophoroside. This anthocyanin was the only one detected in the methanolic extract of the *T. majus* flowers and could be, at least in part, responsible for the typical colour of this flower, as has been described for other flowers [32,42,43]. Peak 12 had a UV spectrum at λ_max_ 355 nm similar to that of the standard quercetin-3-*O*-rutinoside, with λ_max_ at 356 nm, which indicates that this compound is a quercetin derivative. The MS analysis revealed the presence of an [M-H]^−^ ion at *m*/*z* 505 and fragment ions at *m*/*z* 301 and 463. The first fragment corresponds to the loss of the acetylhexosyl moiety [M-H-204]^−^ and the second fragment to the acetyl moiety [M-H-42]^−^. Peak 12 was thus tentatively identified as quercetin-3-*O*-acetylhexoxide. Peaks 13 and 14 had UV spectra at λ_max_ 348, indicating that both are kaempferol derivatives [44]. Both peaks had an MS^2^ fragment ion at *m*/*z* 285; however, peak 13 had an [M-H]^−^ at *m*/*z* 447, corresponding to kaempferol-3-*O*-hexoside, and peak 14 at *m*/*z* 489, indicating the presence of an acetyl unit, kaempferol-3-*O*-acetylhexoside.

Flowers of *T. erecta* were also analysed by HPLC-DAD-ESI-MS^n^, and the phenolic compounds identified were mainly flavonol-glycosides (1603 µg/g, data not shown) based on laricitrin, mirycetin and isorhamnetin (Figure 1 and Figure 2, Table 4). Two anthocyanin-glycosides based on cyanidin and delphinidin, respectively, were also characterised (33 mg/g, data not shown) (Figure 1, Table 4). In addition, one gallotannin and ellagic acid were identified in *T. erecta* flowers (9.20 and 7.40 µg/g, respectively, data not shown) (Figure 1, Table 4). Peak 1 had a UV spectrum at λ_max_ 277 nm, and the MS analysis produced an [M-H]^−^ ion at *m*/*z* 797 and two fragment ions at *m*/*z* 645 [M-H-152]^−^ and 493 [M-H-152]^−^, corresponding to the loss of galloyl moieties, which suggests that this peak is a gallotannin-like compound. Peaks 2, 6, 8, 9 and 12 had similar UV spectra around λ_max_ 354 nm and a common fragment ion in the MS^2^ analysis at *m*/*z* 331 [M-H-162]^−^ releasing the fragment corresponding to laricitrin aglycone, as previously reported in *Malva sylvestris* [45]. Peaks 6, 8 and 9 all had the same [M-H]^−^ ion at *m*/*z* 493 and the same fragment ion at *m*/*z* 331, suggesting that these peaks are isomeric forms of laricitrin-hexoside because of the different retention time. These peaks were the main compounds detected in *T. erecta*. Peaks 3 and 4 had similar UV spectra around λ_max_ 517, corresponding to anthocyanin glycosides. The MS analysis showed that peak 3 had an [M]^+^ at *m*/*z* 611 and yielded MS^2^ fragment ions at *m*/*z* 449 [M-162]^+^ and 287 [M-162]^+^, thus, this peak was tentatively characterised as cyanidin-dihexoside. Peak 4 had an [M]^+^ at *m*/*z* 465 and yielded MS^2^ ions at *m*/*z* 303 [M-162]^+^, corresponding with the loss of a hexosyl moiety—which indicates that this peak is delphinidin-3-*O*-hexoside. Peaks 5 and 10 had similar UV spectra around λ_max_ 358 nm. The MS analysis showed that peak 5 had an [M-H]^−^ at *m*/*z* 479 and an MS^2^ fragment at 317 [M-H-162]^−^, showing that this peak is myricetin-3-*O*-hexoside. Peak 10 had an [M-H]^−^ at *m*/*z* 317 and fragment ions at *m*/*z* 299, 271 and 167, indicating that it is the aglycone of myricetin. Peak 7 had a characteristic UV spectrum at λ_max_ 365 nm and an [M-H]^−^ at *m*/*z* 301, while MS^2^ yielded ions at *m*/*z* 257 and 229. This fragmentation pattern and the absorbance spectrum identified this peak as ellagic acid. The MS^2^ analysis allowed us to distinguish between ellagic acid and quercetin since both compounds produce an identical [M-H]^−^ ion at *m*/*z* 301 [35]. In addition, the UV and mass spectra of these peaks were compared with those of commercial standards. To the best of our knowledge, this is the first time that ellagic acid has been detected in edible flowers, which is noteworthy because of the beneficial effects on human health described for this compound [45]. Finally, peak 11 was the last flavonol detected in *T. erecta* flowers, with an [M-H]^−^ at *m*/*z* 477 and a fragment ion in MS^2^ at *m*/*z* 315 ([M-H-162]^−^)—corresponding to the loss of the hexoxyl moiety—which indicates that this peak is isorhamnetin-3-*O*-hexoside.

It is remarkable that no hydroxycinnamic acid derivatives were detected in *T. erecta*, in contrast to the other flowers analysed. However, *T. erecta* was the only species where hydrolysable tannins and ellagic acid were detected. In addition, quercetin and kaempferol derivatives were not detected, unlike in *T. majus*, and only laricitrin derivatives and myricetin derivatives were characterised as the main flavonols in *T. erecta* (Figure 1, Table 4).

The phenolic compounds in flowers of *S. oleracea* were also characterised mainly as flavonol-glycosides (277.7 µg/g, data not shown) based on quercetin (Figure 1 and Figure 2, Table 4). In addition, minor compounds such as anthocyanins and hydroxycinnamic acid derivatives (13.5 and 33.6 µg/g, respectively, data not shown) were identified in the *S. oleracea* flowers (Figure 1, Table 4). Peaks 1, 3–7, 9–11, 13 and 14 displayed similar UV spectra at λ_max_ around 354 nm, and the MS^2^ spectra showed a common fragment at *m*/*z* 301—indicating that these peaks are quercetin derivatives. These peaks released fragment ions corresponding to the loss of rhamnosyl ([M-H-146]^−^), hexoxyl ([M-H-162]^−^), rutinosyl ([M-H-308]^−^) and glucuronyl ([M-H-176]^−^) moieties. Peaks 10, 11, 13 and 14 were identified as quercetin acetylhexoside derivatives by the loss of −162 and −42 amu, corresponding to hexoxyl and acetyl residues, respectively. Peak 2 was positively characterised according to its retention time and UV and mass spectra, by comparison with the commercial standard, and it had an [M]^+^ at *m*/*z* 449 and an MS^2^ fragment ion at *m*/*z* 287, showing it to be cyanidin-3-*O*-glucoside. Peak 8 displayed an [M]^+^ at *m*/*z* 479 and an MS^2^ fragment at *m*/*z* 303, which indicates that this peak is delphinidin-3-*O*-glucoside. Peak 12 had a UV spectrum with λ_max_ at 330 nm, an [M-H]^−^ ion at *m*/*z* 515 and an MS^2^ fragment at *m*/*z* 353 from the loss of one of the caffeoyl moieties [M-H-caffeoyl]^−^, which indicates the presence of a dicaffeoylquinic acid [38,40,46]. Peak 15 had an [M-H]^−^ ion at *m*/*z* 677 and MS^2^ fragment ions at *m*/*z* 515 and 353; its MS^2^ spectra, which correspond to the losses of the caffeoylquinic ([M-H-353]^−^) and hexosyl ([M-H-162]^−^) moieties, suggest that it is a caffeoylquinic acid dihexose derivative.

### 2.3. Antioxidant Capacity

The antioxidant capacity was determined using two assays Oxygen Radical Absorbance Capacity (ORAC) and Trolox Equivalent Antioxidant capacity (TEAC), exhibiting a similar trend in both. The greatest antioxidant capacites in both the ORAC and TEAC assays were found for *T. erecta*, followed by *T. majus* and *S. oleracea*. It is quite surprising that the ORAC values showed a large variation, from 10.77 µmol TE/g in *S. oleracea* to 266.07 µmol TE/g in *T. erecta* (Table 3). The TEAC values also varied widely, from 9.51 to 66.16 µmol TE/g in *S. oleracea* and *T. erecta,* respectively (Table 3)*.* Our results are in line with those described in the scientific literaturefor other edible flowers [10,47,48]. The results of the two antioxidant assays were highly correlated (*r* = 0.972, *p* < 0.01, Table 5), but the ORAC values were around four-fold higher than the TEAC values; hence, they reflect different antioxidant activities. The ORAC assay measures specifically the ability of compounds to scavenge oxygen free radicals and, as such, is considered the closest to human physiology [30], whereas TEAC method provide information about the capacity of an extract to scavenge free radicals with different chemical structures. In addition, the differences in the antioxidant capacity estimations could be due to the fact that the ORAC assay, overestimates the capacity of antioxidants with low reactivity [49]. However, this method is widely used at present for pure compounds, foods, botanicals, nutraceuticals and commercial products. Similar results have been reported for different plant foods when the antioxidant capacity is analysed by different assays [50,51].

**Table 5:** Pearson’s correlation coefficients (*r*) and their statistical significance for the correlations of total phenolic compounds (TPC) and individual phenolic compounds determined by HPLC-DAD-ESI/MS^n^ with the oxygen radical absorbance capacity (ORAC) and Trolox equivalent antioxidant capacity (TEAC).

Parameters	*r_ORAC_*	*p*	*r_TEAC_*	*p*
TPC	0.865	<0.05	0.931	<0.05
Total anthocyanins	−0.182	ns	−0.259	ns
Total flavonols	0.976	<0.01	0.987	<0.01
Total hydroxycinnamic acids	−0.596	ns	−0.666	ns
Hydrolysable tannins	0.971	<0.01	0.998	<0.01
Ellagic acid	0.971	<0.01	0.998	<0.01
Σ Individual phenolics	0.953	<0.01	0.952	<0.01

ns, not significant.

Despite the differences between the ORAC and TEAC assays, both showed positive and significant correlations (*p* < 0.05) with the content of TPC (*r* = 0.865 and *r* = 0.931, respectively, Table 5), indicating that the phenolic compounds are major contributors to the high antioxidant capacity, as previously reported for other edible flowers [1,7,10,33,34,48]. Taking into consideration the individual phenolic compounds, the flavonols, hydrolysable tannins and ellagitannins—as well the sum of the identified phenolic compounds—showed significant (*p* < 0.01) and positive correlations (*r* values > 0.9) with the ORAC and TEAC antioxidant capacities (Table 5). However, no relationships were observed between the antioxidant capacity and the contents of anthocyanins and hydroxycinnamic acids (Table 5). Hence, the radical scavenging activity of the extracts depended on the total amount of phenolics, but was determined also by the main compounds identified in each species. *Tagates erecta* showed the highest antioxidant capacity as well as the highest amounts of TPC and flavonols. Since hydroxycinnamic acids were not identified and anthocyanins were found at very low levels in these samples (Figure 2, Table 4), flavonols appear to be the major contributor to the antioxidant capacity, as described by other authors [34]. In addition, the high antioxidant capacity of *T. erecta* could be explained, at least in part, by the presence of ellagic acid in this species (Figure 2, Table 4), in agreement with previously published reports in which the antioxidant activity of this compound was evaluated [52]. The antioxidant capacity of *S. oleracea* could also be explained by the TPC and the proportion of flavonols, in both cases higher than in *T. majus*. The lowest antioxidant capacity was found for *S. oleracea*, which might be due to the fact that the flowers of this species had the lowest concentrations of TPC and flavonols and the highest proportions of anthocyanins and hydroxycinnamic acids (Figure 2, Table 4)—compounds that were not associated with the antioxidant capacity, as described above (Table 5).

Although the edible flowers of these three species possess an antioxidant capacity, it should be borne in mind that this activity measured *in vitro* cannot be extrapolated simply to the *in vivo* situation, because bioavailability, metabolism and biotransformation as well as chemical reactivity are important in the determination of the *in vivo* capacity [53].

## 3. Experimental Section

### 3.1. Samples

The fresh edible flowers of three species, monks *cress* (*Tropaeolum majus*), *marigold* (*Tagetes erecta*) *and paracress* (*Spilanthes oleracea*) (Figure 2), were provided by the company “Alba-Soldevila” (Alguarie, Lleida, Spain). The flowers were stored at 6 °C during transport and immediately after arrival at the laboratory. For nutritional analysis, the flowers were used fresh—whereas for the TDF, phenolic compounds and total antioxidant capacity assays, samples were freeze-dried and stored at 4 °C in dry conditions until analysis.

### 3.2. Standards and Reagents

The Folin-Ciocalteu’s phenol and ABTS reagent were supplied by Sigma Aldrich (St. Louis, MO, USA) and the chemical solvents (trifluoroacetic acid, ethanol, methanol, acetonitrile, formic acid, acetic acid, ammonium acetate, ammonium hydroxide and milli-Q water) were obtained from Panreac (Barcelona, Spain). All chemicals were at least high-performance liquid chromatography (HPLC) grade quality. Cyanidin-3-*O*-glucoside was a generous gift from Cristina García-Vigueras, punicalagin and ellagic acid were purchased from LGC Standards (Barcelona, Spain), and gallic acid, chlorogenic acid, quercetin-3-*O*-rutinoside and Trolox were from Sigma Aldrich (St. Louis, MO, USA).

### 3.3. Proximate and Mineral Composition

The proximate composition (moisture, total solids, protein, fat, ash and carbohydrates) was analysed by following the Association of Official Analytical Chemists (AOAC) official methods [54]. The moisture and total solids contents were obtained by drying the flowers in an oven at 110 °C until constant weight was achieved. The crude protein content of samples was estimated by the macro-Kjeldahl method (N × 6.25). The total fat was determined using a Soxhlet procedure. The ash content was quantified after incineration of the samples at 525 °C for 24 h. The total carbohydrates were calculated by difference, whereas the total energy was obtained according to the Atwater number. Total dietary fibre (TDF) was determined by following the enzymatic and gravimetric method described by Prosky *et al*. [55]. The samples were digested consecutively with α-amylase (thermo-stable), protease and amyloglucosidase to obtain the residue resistant to *in vitro* intestinal digestion. The chemical compounds of the dietary fibres were precipitated by adding 90% ethanol; after one hour, samples were filtered through glass filters using a Fibertec System E 1023 (Högänas, Sweden). The residues were desiccated overnight and then weighed to determine the residue amount. The protein and ash contents were analysed in the residues to eliminate the amounts of these compounds and to obtain the final weight of the residue, which was expressed as percentage of total dietary fibre (TDF). The mineral composition was analysed by inductively coupled plasma optical emission spectroscopy (ICP-OES), using an ICAP 6500 Duo Thermo model (Thermo Scientific, Cambridge, UK), after microwave-assisted digestion (UltraCLAVE, Milestone Inc., Shelton, CT, USA) with H_2_O_2_ and HNO_3_ (1:4 *v*/*v*). All data related to the nutritional composition of the flowers were expressed on a fresh weight basis.

### 3.4. Preparation of the Flower Extracts

Flower extracts were used for the analysis of total and individual phenolic compounds and for the hydrophilic antioxidant capacity. To obtain the extracts, 100 mg samples of the lyophilised flowers were extracted separately in covered Erlenmeyer flasks with 25 mL of acidified ethanol (80% ethanol, 19% H_2_O and 1% 0.1% trifluoroacetic acid, *v*/*v*/*v*) at room temperature for 24 h, on an orbital shaker. To minimise compound oxidation, the solutions were purged with nitrogen and the extraction was carried out in the dark. After this, the extracts were filtered through 0.45 µm filters and concentrated under reduced pressure at 30 °C. Then, the extracts were resuspended in 3 mL of water and passed through a C_18_-SPE column (Waters, Milford, MA, USA), previously activated with 10 mL of methanol followed by 20 mL of water. The cartridge was washed with 20 mL of water and compounds of interest were eluted with 10 mL of methanol. Immediately, the eluted volumes were evaporated and lyophilised to a dry powder, which was resuspended in methanol in a volumetric flask.

### 3.5. Total Phenolic Compounds

The total phenolic compounds (TPC) in the flowers were analysed using Folin-Ciocalteu’s colorimetric assay as described by Singleton and Rossi [56], with minor modifications. A volume of 1 mL of the sample was mixed with 7.5 mL of distilled water and 0.3 mL of Folin-Ciocalteu’s phenol (diluted 1:10) and allowed to react for three minutes. Then, 1 mL of a saturated solution of Na_2_CO_3_ was added and allowed to react for 20 min at room temperature. The absorbance was measured at 760 nm with a UV-VIS spectrophotometer (Evolution 300, Thermo Scientific, Cambridge, UK) and the TPC concentration of the samples was expressed as mg of gallic acid equivalents (GAE)/g of fresh weight.

### 3.6. Hydrophilic Antioxidant Capacity Assays

The hydrophilic antioxidant capacity was determined in the flower extracts, obtained as described above, using two procedures: Trolox Equivalent Antioxidant Capacity (TEAC assay) and Oxygen Radical Absorbance Capacity (ORAC assay).

#### 3.6.1. TEAC Assay

The TEAC assay [57] was based on the reduction of the ABTS radical action by the antioxidants present in the samples. The ABTS [2,2'-azino-bis(3-ethylbenzothiazoline-6-sulphonic acid)] was prepared by passing ABTS, dissolved in 5 mM phosphate buffered saline (PBS), through manganese dioxide on a filter paper. This solution was diluted in 5 mM PBS (pH 7.4) to an absorbance of 0.70 (±0.02) at 734 nm, measured using a UV-VIS spectrophotometer (Evolution 300, Thermo Scientific; UK). Trolox was used as the antioxidant standard, and the results were expressed as µmol of Trolox equivalents (TE)/g of fresh weight.

#### 3.6.2. ORAC assay

The ORAC assay is a fluorescence method used widely to assess the antioxidant capacity in biological samples. It is based on the inhibition of a peroxy-radical-induced oxidation initiated by the thermal-based decomposition of azo compounds such as 2,2'-azobis(2-amidino-propane) dihydrochloride (AAPH), using fluorescein as a fluorescent probe and Trolox as a standard substrate [58]. This assay was carried out with a fluorescent microplate reader (Synergy 2 Multi-Mode Microplate Reader, Winooski, VT, USA) and 96-well black microplates. Fluorescence filters with an excitation wavelength of 485 nm and an emission wavelength of 520 nm were used. A stock fluorescein solution (Stock #1) was prepared by dissolving 0.0225 g of fluorescein (FL) in 50 mL of 0.075 M phosphate buffer (pH 7.0). A second stock solution was prepared by diluting 50 µL of stock solution #1 in 10 mL of phosphate buffer. An 800-µL portion of solution #2 was mixed with 50 mL of phosphate buffer; of this, 200 µL were added to each well. A stock standard of Trolox (500 µM) was aliquoted into small vials for storage at −80 °C until use. In the standard assay, 20 µL of the Trolox calibration solutions (6.25, 12.5, 25, 50 µM) in phosphate buffer (0.075 M, pH 7.0) were pipetted into the appropriate wells. Each day, a new set of stock Trolox vials was removed from the freezer for use. In the sample assay, 20 µL of each diluted sample extract were pipetted into the appropriate well. The same volume of water was pipetted for the blanks. The plate reader was equipped with an incubator and two injection pumps; the temperature of the incubator was set to 37 °C. The rate of peroxyl radical production from AAPH is temperature sensitive, so the timing and handling of the AAPH solution are critical. Thus, a new AAPH solution was prepared for each run. The old FL and AAPH solutions were flushed from the syringes, which were then primed with new FL and AAPH before starting the next run. The instrument pipetted 200 µL of FL from pump #1 into the respective wells. After incubating for 15 min at 37 °C, pump 2 injected 20 µL of AAPH into the respective wells. The plate contents were mixed by shaking for 8 s following each injection and/or reading; the readings were initiated immediately. The fluorescence of each well was measured from the bottom every 60 s for 90 min. The results were calculated as described by Prior *et al.* [59] and expressed as µmol of TE/g of fresh weight.

### 3.7. Analysis of Phenolic Compounds by HPLC-DAD and HPLC-DAD-ESI-MS^n^

Methanolic extracts of the flowers were analysed on an HPLC system (Waters 2695) equipped with a diode array detector (Waters 2996), scanning from 200 to 600 nm. Separation of the different phenolic compounds was performed using a LiChroCART RP-18 column (250 × 4.6 mm i.d., 5 μm) with a pre-column (4 × 4 mm i.d.) of the same material (Merck, Darmstadt, Germany). The mobile phases used were 4.5% aqueous formic acid (solvent A) and acetonitrile (solvent B), at a flow rate of 0.8 mL/min. Elution began with a linear gradient from 0% to 30% B in 70 min, followed by washing and then a return to the initial conditions. Chromatograms were recorded at 280, 320, 360 and 520 nm. The samples were also analysed using an LC-MSD-Trap VL-01036 liquid chromatograph-ion trap mass spectrometer (Agilent Technologies, Waldbronn, Germany), equipped with an electrospray ionization (ESI) source operating in positive mode for anthocyanins and in negative mode for all other compounds to confirm each peak identity. The nebulizer gas was nitrogen; the pressure and the flow rate of the drying gas were set at 65 psi and 9.5 L/min, respectively. Analyses were carried out using full-scan and data-dependent MS^2^ scanning from *m*/*z* 100 to 1500. Collision-induced fragmentation experiments were performed in the ion trap using helium as the collision gas, and the collision energy was set at 30%. The heated capillary and voltage were maintained at 350 °C and 4 kV, respectively. The chromatographic separations were performed in the same column and pre-column, with 1% aqueous formic acid and acetonitrile as the mobile phases and using the same gradient, as detailed above. Phenolic compounds detected in the samples were characterised according to their UV and mass spectra, their retention times—by chromatographic comparisons with authentic standards, when available—and their absorbance spectra and MS^2^ fragmentation, based on previously reported data [35,36,37,38,39,40]. Anthocyanins were quantified from their chromatographic peak areas recorded at 520 nm and expressed as cyanidin-3-*O*-glucoside equivalents, hydroxycinnamic acid derivatives at 320 nm as chlorogenic acid equivalents, flavonol conjugates at 360 nm as quercetin-3-*O*-rutinoside, hydrolysable tannins at 280 nm as gallic acid equivalents, and ellagic acids at 360 nm.

### 3.8. Statistical Analysis

All determinations were carried out in triplicate and the data were expressed as the mean ± standard deviation. The data were analysed by the SPSS Statistical Package, version 19.0 for Windows (IBM, Madrid, España). An analysis of variance was included in the data treatment to determine the differences in the analysed parameters as a function of the sample, and Tukey’s test was applied as a *post*-*hoc* test to determine the differences among means. Relationships between variables were examined using Pearson correlation coefficients. *p* values <0.05 were considered statistically significant.

## 4. Conclusions

Edible flowers are becoming increasingly popular in the human diet, mainly due to improvements in the organoleptic properties of different dishes and foodstuffs. For this reason it is very important to know their nutritional composition as well as other functional and beneficial properties related to their phenolic compounds and antioxidant properties. In general, this potential beneficial effect regarding human health differed among the three species and depended on the content of total phenolic compounds and the proportions of the different phenolic groups. These results suggest that edible flowers could be a natural source of polyphenols for functional foods, but their real contribution to the overall *in vivo* antioxidant activity is still under discussion.
